# Diphtheria &c.

**Published:** 1889-03

**Authors:** J. W. Duncan

**Affiliations:** Georgia


### DIPHTHERIA COMPLICATED WITH SCARLATINA.

BY J. W. DUNCAN, M. D., OF GEORGIA.

Read before the Atlanta Medical Society, Feb 1889.

Nov. 6, 1888, I was called to see H. M., a little girl aged 5 years. I learned that she had been quite sick since Nov. 2nd. Diagnosis, diphtheria complicating scarlatina.

The temp. was 104°; pulse too frequent to be timed. The physical signs were were well marked. General diffused scarlatinous eruption. Tongue heavily furred, with the papillæ prominent, producing the strawberry appearance. Exudation over pharynx, soft palate, uvula and in nasal passages, completely blocking up the nose, so that the child had to breathe through the mouth. The larynx was also involved, as the child could not speak except in a whisper. Both eyes were influenced, and there was diphtheretic exudation, especially on the lids of the left eye, and on Nov. 10th the globe of the left eye became involved, and in a few hours there was entire loss of vision in that eye, the eye being of a grayish hue and showing an uneasiness like a hernia or staphyloma near the pupil, at which point the tunics gave way, and the aqueous humor escaped.

The child had complained of severe earache, but was at this time (Nov. 6th to 9th) so delirious that she did not recognize her mother. There was great difficulty in respiration. After a few days the fever declined, the membranes were detached and ceased to reform.

The nasal passages, from which an abundance of membrane had been discharged were so much improved that she could again breathe through the nose.

The delirium had disappeared, and it seemed that convalescence was fully established.

She was taking milk with a little brandy and bovinine with relish. The eruption had faded, but about the 14th or 15th day of the sickness there was a slight relapse, with a return of fever to 102°, and also a general scarlatenous rash, which lasted 4 or 5 days. During this time the lids of the lost eye became very much inflamed and enormously swollen. The lids were very much improved in 24 hours, but in two or three days the lower lid became more inflamed again, and in a few days it became necessary to lance it, and about a half an ounce of pus was discharged at once. The pus continued to form rather freely for two weeks, and it did not entirely cease to form until the end of the fourth week. Pus was discharged from both ears during the third and fourth week.

Desquamation began at the 1st of the third week, and by the end of the fourth week the child had literally peeled off. During the third and fourth week it had a good appetite, appropriated its nourishment well, and could sit up for several hours at a time.

During the second week there was considerable albumen in the urine, but it was not abundant except for a few days.

Treatment.—

℞ F. E. jaborandi, gtts. xxx, Tinct. belladon., gtts. xl, F. E. gelsemium, gtts. xl, Spts. niter dulc. ʒi, Syr. simplex, grs. ℥ij.

Sig. One teaspoonful every three hours.

℞ Salicylate quinia, grs. xvi, Antifebrin, grs. v, Flour sulphur, grs. xxx, ft. charts No. 8. Sig. One every 4 hours.

Pinus canadensis, con., ʒiv, Glycerine, qs. ℥ii. Sig. ¼ teaspoonful every 1 or 2 hours, and also use the same diluted with warm water, with syringe in nose.

Later, I used the $\frac{1}{5000}$ % bichloride solution in the nose with syringe every 2 or 3 hours.

℞ Tinct. iodine com., gtts. xxx; Tinct. ferri chlor. gtts. L; Kali chloras, grs. xxx; Aqua cinnamni ʒ iv; Glycerine, qs. ℥ ii. Sig. One teaspoonful every 2 hours.

*For eyes:* ℞ Sulph. atrophia, grs. ss; Sulph. morphia, grs. iv; Acid boracic, grs. vi; Aqua dist. qs. ℥ i. Put a few drops in eye every 3 hours.

Nov. 8th I substituted the following for Nos. 1 and 2:

℞ Tinct. digitalis, gtts. xxx; Tinct. strophanthus, gtts. xxv.; F. E. Jaborandi, gtts. xxx; Salicylate quinia, grs. xv; Syr. simplex, qs. ℥ ii. Sig. One teaspoonful every 3 hours.

I had the child's body anointed with salty lard frequently.

I had turpentine vapor generated quite freely in the room.

Gave 15 drops of brandy in sweet milk every 2 or 3 hours.

The medicines were alternated and there being several formulas, the space of time between doses was short, which I consider an important item in the treatment of diphtheria.

The case was under close observation for more than a month. I saw her regularly until the 14th of Dec. On Christmas day I called to see how she was getting along. I think that she was as happy a child as I saw that day. I have seen her only once since. She was hearty and quite playful.

After reviewing the case and the treatment, I am at a loss to know whether I could have omitted any of the remedies without detriment to my patient. I have great confidence in the iodine, iron and potash treatment. I consider pinus canadensis and glycerine one of the best local remedies.

I believe that the bichloride did invaluable service in this case. I also believe that the jaborandi, digitalis and strophanthus were almost indispensable.

